# 4-Bromo-*N*-(diethyl­carbamothio­yl)­benzamide

**DOI:** 10.1107/S1600536809003183

**Published:** 2009-01-31

**Authors:** Gün Binzet, Ulrich Flörke, Nevzat Külcü, Hakan Arslan

**Affiliations:** a Department of Chemistry, Faculty of Arts and Sciences, Mersin University, Mersin TR 33343, Turkey; b Department of Chemistry, University of Paderborn, Paderborn D33098, Germany; c Department of Natural Sciences, Fayetteville State University, Fayetteville, NC 28301, USA; d Department of Chemistry, Faculty of Pharmacy, Mersin University, Mersin, TR 33169, Turkey

## Abstract

The synthesis of the title compound, C_12_H_15_BrN_2_OS, involves the reaction of 4-bromo­benzoyl chloride with potassium thio­cyanate in dry acetone, followed by condensation of 4-bromo­benzoyl isothio­cyanate with diethyl­amine. The carbonyl and thio­carbonyl bond lengths indicate that these correspond to double bonds. The short C—N bond lengths reveal the effects of resonance in this part of the mol­ecule. The conformation of the mol­ecule with respect to the thio­carbonyl and carbonyl units is twisted, with torsion angles of −5.7 (3) and 87.2 (2)°. The N atom of the diethyl­amine group is *sp* ^2^-hybridized: the sum of the angles around the N atom is 359.97 (14)°. The two diethyl groups are twisted in + and − anti­periplanar conformations with angles of −179.89 and 179.92°. In the crystal structure, the mol­ecules form infinite chains *via* an inter­molecular N—H⋯O inter­action.

## Related literature

For the synthesis, see: Özer *et al.* (2009 ▶); Arslan, Flörke & Külcü (2003 ▶), and references therein. For general background, see: Koch (2001 ▶); El Aamrani *et al.* (1998 ▶, 1999 ▶); Arslan *et al.* (2006 ▶); Arslan, Flörke & Külcü (2007 ▶); Arslan, Flörke, Külcü & Binzet (2007 ▶); Yuan *et al.* (2001 ▶); Zhang *et al.* (2004 ▶); Weiqun *et al.* (2004 ▶). For related compounds, see: Arslan, Külcü & Flörke (2003 ▶); Arslan *et al.* (2004 ▶); Khawar Rauf *et al.* (2009*a* ▶,*b* ▶); Khawar Rauf, Bolte & Anwar (2009 ▶); Khawar Rauf, Bolte & Rauf (2009 ▶). For bond-length data, see: Allen *et al.* (1987 ▶).
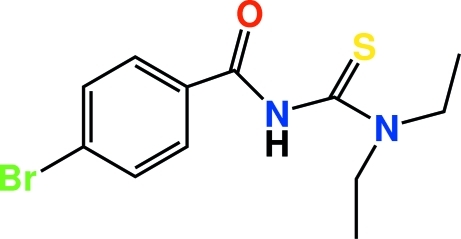


## Experimental

### 

#### Crystal data

C_12_H_15_BrN_2_OS
*M* *_r_* = 315.23
Monoclinic, 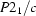

*a* = 6.9955 (9) Å
*b* = 18.680 (2) Å
*c* = 10.0816 (13) Å
β = 95.361 (3)°
*V* = 1311.7 (3) Å^3^
*Z* = 4
Mo *K*α radiation
μ = 3.28 mm^−1^
*T* = 120 (2) K
0.38 × 0.37 × 0.11 mm

#### Data collection

Bruker SMART APEX diffractometer
Absorption correction: multi-scan (*SADABS*; Bruker, 2002 ▶) *T*_min_ = 0.329, *T*_max_ = 0.714
10831 measured reflections
3117 independent reflections
2730 reflections with *I* > 2σ(*I*)
*R*_int_ = 0.027

#### Refinement

*R*[*F* ^2^ > 2σ(*F* ^2^)] = 0.025
*wR*(*F* ^2^) = 0.064
*S* = 1.06
3117 reflections
158 parameters
1 restraint
H atoms treated by a mixture of independent and constrained refinement
Δρ_max_ = 0.59 e Å^−3^
Δρ_min_ = −0.26 e Å^−3^

### 

Data collection: *SMART* (Bruker, 2002 ▶); cell refinement: *SAINT* (Bruker, 2002 ▶); data reduction: *SAINT*; program(s) used to solve structure: *SHELXTL* (Sheldrick, 2008 ▶); program(s) used to refine structure: *SHELXTL*; molecular graphics: *SHELXTL*; software used to prepare material for publication: *SHELXTL*.

## Supplementary Material

Crystal structure: contains datablocks I, global. DOI: 10.1107/S1600536809003183/at2713sup1.cif

Structure factors: contains datablocks I. DOI: 10.1107/S1600536809003183/at2713Isup2.hkl

Additional supplementary materials:  crystallographic information; 3D view; checkCIF report

## Figures and Tables

**Table 1:** Hydrogen-bond geometry (Å, °)

*D*—H⋯*A*	*D*—H	H⋯*A*	*D*⋯*A*	*D*—H⋯*A*
N1—H1⋯O1^i^	0.896 (5)	2.016 (9)	2.882 (2)	162 (2)

Symmetry code: (i) 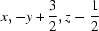.

**Fig. 1.**
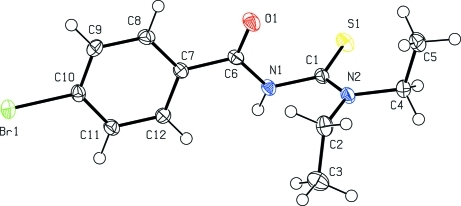
The molecular structure of (I). Displacement ellipsoids are drawn at the 50% probability level.

**Fig. 2.**
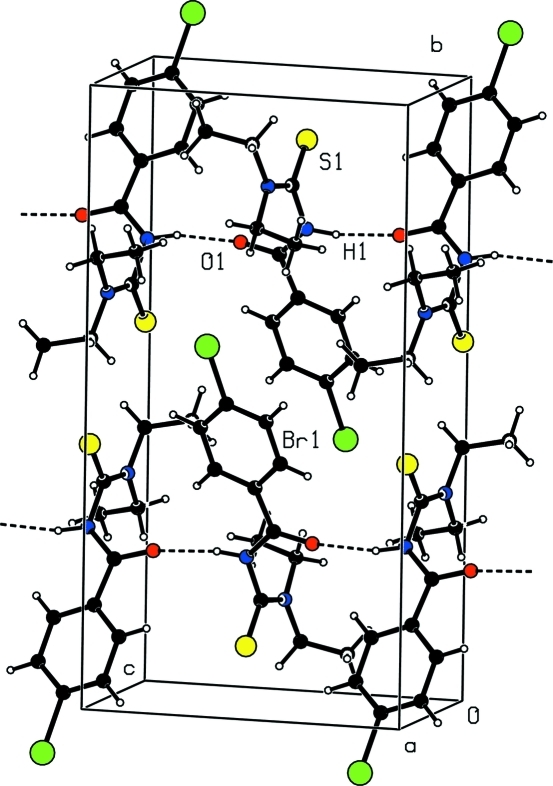
Part of the structure showing the formation of endless chains involving N—H···O hydrogen bonds.

## supplementary crystallographic information

### Comment

Transition metal complexes bearing thiourea ligand or its derivatives have been one of the highlights in coordination chemistry, which are used as reactant for extraction of some transition metal ions (Koch, 2001; El Aamrani *et al.*, 1998, 1999). Moreover, the growing interest for thiourea derivative ligands and their metal complexes result from the important role they play in biological systems (Yuan *et al.*, 2001; Zhang *et al.*, 2004; Weiqun *et al.*, 2004).

Recently, our research has focussed on the chemical and physical properties of thiourea derivatives and their metal complexes (Arslan *et al.*, 2006; Arslan, Flörke & Külcü, 2007; Arslan, Flörke, Külcü & Binzet, 2007). In the present work, we report the crystal structure of 4-bromo-*N*-(diethylcarbamothioyl)benzamide, (I). The molecular structure of the title compound is depicted in Fig. 1.

The typical thiourea carbonyl [C6—O1 = 1.230 (2) Å] and thiocarbonyl (C1—S1 = 1.6638 (18) Å) double bonds as well as shortened C—N bond lengths (C1—N1 (1.435 (2) Å), C1—N2 (1.325 (2) Å) and C6—N1 (1.355 (2) Å)) are observed in the title compound. These bond lengths in the title compound are comparable to those of related structures (Khawar Rauf *et al.*, 2009*a*,*b*; Khawar Rauf, Bolte & Anwar, 2009; Khawar Rauf, Bolte & Rauf, 2009; Arslan, Flörke & Külcü, 2003; Arslan *et al.*, 2004). The other bond lengths in (I) show normal values (Allen *et al.*, 1987).

The conformation of the title molecule with respect to the thiocarbonyl and carbonyl moieties is twisted, as reflected by the C1—N1—C6—O1, C6—N1—C1—N2, and C6—N1—C1—S1 torsion angles of -5.7 (3) °, 87.2 (2) ° and -94.57 (18) °, respectively. The dihedral angle between the 4-bromophenyl ring and the plane O1/N1/C7/C6 is 10.10 (3) °, and the dihedral angle between the 4-bromophenyl ring and the plane S1/C1/N1/N2 is 86.98 (3) °. The atom N2 is *sp*^2^-hybridized, because of the sum of the angles around atom N2 is 359.97 (14) °. The two diethyl groups are twisted in a + and - antiperiplanar conformation with -179.89 ° and 179.92 °.

Iintermolecular N—H···O (*x, -y+1.5, z-0.5*) hydrogen bonds (Table 1) link the molecules into endless chains, as shown in Fig. 2.

### Experimental

The title compound was prepared with a procedure similar to that reported in the literature (Arslan, Külcü & Flörke, 2003; Özer *et al.*, 2009). A solution of 4-bromobenzoyl chloride (0.01 mol) in acetone (50 cm^3^) was added dropwise to a suspension of potassium thiocyanate (0.01 mol) in acetone (30 cm^3^). The reaction mixture was heated under reflux for 30 min, and then cooled to room temperature. A solution of diethylamine (0.01 mol) in acetone (10 cm^3^) was added and the resulting mixture was stirred for 2 h. Hydrochloric acid (0.1 N, 300 cm^3^) was added to the solution, which was then filtered. The solid product was washed with water and purifed by recrystalization from an ethanol:dichloromethane mixture (1:2). Analysis calculated for C_12_H_15_N_2_OSBr: C 45.7, H 4.8, N 8.9%. Found: C 45.7, H 4.9, N 8.7%.

### Refinement

H atoms bound to C atoms were placed geometrically and allowed to ride on their parent atoms, with C—H = 0.95-0.99 Å and *U*_iso_(H) = 1.2 or 1.5 *U*_eq_(C). The nitrogen-bound H atom was located in a difference Fourier map and refined freely.

### Crystal data

**Table:**

C_12_H_15_BrN_2_OS	*F*(000) = 640
*M**_r_* = 315.23	*D*_x_ = 1.596 Mg m^−^^3^
Monoclinic, *P*2_1_/*c*	Mo *K*α radiation, λ = 0.71073 Å
Hall symbol: -P 2ybc	Cell parameters from 891 reflections
*a* = 6.9955 (9) Å	θ = 2.3–28.2°
*b* = 18.680 (2) Å	µ = 3.28 mm^−^^1^
*c* = 10.0816 (13) Å	*T* = 120 K
β = 95.361 (3)°	Prism, colourless
*V* = 1311.7 (3) Å^3^	0.38 × 0.37 × 0.11 mm
*Z* = 4	

### Data collection

**Table:**

Bruker SMART APEX diffractometer	3117 independent reflections
Radiation source: sealed tube	2730 reflections with *I* > 2σ(*I*)
graphite	*R*_int_ = 0.027
φ and ω scans	θ_max_ = 27.9°, θ_min_ = 2.2°
Absorption correction: multi-scan (*SADABS*; Bruker, 2002)	*h* = −9→7
*T*_min_ = 0.329, *T*_max_ = 0.714	*k* = −24→24
10831 measured reflections	*l* = −13→13

### Refinement

**Table:**

Refinement on *F*^2^	Primary atom site location: structure-invariant direct methods
Least-squares matrix: full	Secondary atom site location: difference Fourier map
*R*[*F*^2^ > 2σ(*F*^2^)] = 0.025	Hydrogen site location: difference Fourier map
*wR*(*F*^2^) = 0.064	H atoms treated by a mixture of independent and constrained refinement
*S* = 1.06	*w* = 1/[σ^2^(*F*_o_^2^) + (0.0328*P*)^2^ + 0.2678*P*] where *P* = (*F*_o_^2^ + 2*F*_c_^2^)/3
3117 reflections	(Δ/σ)_max_ = 0.001
158 parameters	Δρ_max_ = 0.59 e Å^−^^3^
1 restraint	Δρ_min_ = −0.26 e Å^−^^3^

### Special details

**Table:**

Geometry. All esds (except the esd in the dihedral angle between two l.s. planes) are estimated using the full covariance matrix. The cell esds are taken into account individually in the estimation of esds in distances, angles and torsion angles; correlations between esds in cell parameters are only used when they are defined by crystal symmetry. An approximate (isotropic) treatment of cell esds is used for estimating esds involving l.s. planes.
Refinement. Refinement of F^2^ against ALL reflections. The weighted R-factor wR and goodness of fit S are based on F^2^, conventional R-factors R are based on F, with F set to zero for negative F^2^. The threshold expression of F^2^ > 2sigma(F^2^) is used only for calculating R-factors(gt) etc. and is not relevant to the choice of reflections for refinement. R-factors based on F^2^ are statistically about twice as large as those based on F, and R- factors based on ALL data will be even larger.

### Fractional atomic coordinates and isotropic or equivalent isotropic displacement parameters (Å^2^)

**Table:**

	*x*	*y*	*z*	*U*_iso_*/*U*_eq_	
Br1	0.18884 (3)	0.416437 (9)	0.341437 (19)	0.02229 (7)	
S1	0.41112 (7)	0.90370 (2)	0.43285 (5)	0.02424 (11)	
O1	0.4748 (2)	0.73637 (7)	0.62427 (13)	0.0265 (3)	
N1	0.5090 (2)	0.76602 (7)	0.41132 (14)	0.0176 (3)	
H1	0.478 (3)	0.7585 (12)	0.3242 (7)	0.035 (6)*	
N2	0.7563 (2)	0.84313 (7)	0.48486 (15)	0.0184 (3)	
C1	0.5705 (3)	0.83737 (9)	0.44671 (17)	0.0180 (4)	
C2	0.8900 (3)	0.78163 (9)	0.49166 (19)	0.0230 (4)	
H2A	0.8245	0.7391	0.5248	0.028*	
H2B	1.0018	0.7926	0.5561	0.028*	
C3	0.9600 (3)	0.76427 (10)	0.3581 (2)	0.0290 (4)	
H3A	1.0476	0.7233	0.3674	0.043*	
H3B	1.0277	0.8058	0.3258	0.043*	
H3C	0.8501	0.7525	0.2943	0.043*	
C4	0.8433 (3)	0.91336 (9)	0.51931 (19)	0.0219 (4)	
H4A	0.7698	0.9512	0.4681	0.026*	
H4B	0.9764	0.9142	0.4935	0.026*	
C5	0.8459 (3)	0.92934 (10)	0.66664 (19)	0.0265 (4)	
H5A	0.9048	0.9763	0.6855	0.040*	
H5B	0.9204	0.8925	0.7176	0.040*	
H5C	0.7141	0.9296	0.6922	0.040*	
C6	0.4567 (3)	0.72010 (9)	0.50554 (17)	0.0172 (3)	
C7	0.3850 (2)	0.64814 (9)	0.45935 (17)	0.0166 (3)	
C8	0.3120 (3)	0.60335 (10)	0.55234 (18)	0.0197 (4)	
H8A	0.3030	0.6200	0.6406	0.024*	
C9	0.2519 (3)	0.53439 (9)	0.51763 (18)	0.0203 (4)	
H9A	0.2013	0.5039	0.5812	0.024*	
C10	0.2670 (2)	0.51082 (9)	0.38901 (18)	0.0180 (3)	
C11	0.3388 (3)	0.55432 (9)	0.29424 (18)	0.0204 (4)	
H11A	0.3481	0.5372	0.2063	0.025*	
C12	0.3970 (3)	0.62334 (9)	0.32955 (18)	0.0190 (4)	
H12A	0.4453	0.6539	0.2651	0.023*	

### Atomic displacement parameters (Å^2^)

**Table:**

	*U*^11^	*U*^22^	*U*^33^	*U*^12^	*U*^13^	*U*^23^
Br1	0.02598 (11)	0.01446 (10)	0.02654 (11)	−0.00248 (6)	0.00300 (7)	−0.00079 (6)
S1	0.0274 (3)	0.0178 (2)	0.0274 (3)	0.00464 (17)	0.00185 (19)	−0.00387 (17)
O1	0.0459 (9)	0.0198 (6)	0.0141 (6)	−0.0039 (6)	0.0046 (6)	−0.0019 (5)
N1	0.0241 (8)	0.0153 (7)	0.0131 (7)	−0.0018 (6)	0.0007 (6)	−0.0016 (5)
N2	0.0240 (8)	0.0130 (7)	0.0184 (8)	−0.0006 (6)	0.0028 (6)	−0.0016 (5)
C1	0.0277 (10)	0.0139 (8)	0.0127 (8)	−0.0025 (7)	0.0040 (7)	−0.0005 (6)
C2	0.0242 (10)	0.0171 (8)	0.0270 (10)	0.0023 (7)	−0.0025 (8)	0.0019 (7)
C3	0.0293 (11)	0.0264 (10)	0.0313 (11)	0.0090 (8)	0.0034 (8)	−0.0025 (8)
C4	0.0259 (10)	0.0172 (8)	0.0230 (10)	−0.0052 (7)	0.0040 (7)	−0.0023 (7)
C5	0.0317 (11)	0.0246 (9)	0.0228 (10)	−0.0065 (8)	0.0009 (8)	−0.0063 (7)
C6	0.0206 (9)	0.0157 (8)	0.0155 (9)	0.0023 (6)	0.0021 (7)	−0.0007 (6)
C7	0.0174 (8)	0.0150 (7)	0.0172 (9)	0.0017 (6)	0.0007 (7)	−0.0006 (6)
C8	0.0244 (9)	0.0205 (8)	0.0146 (9)	0.0001 (7)	0.0035 (7)	−0.0009 (7)
C9	0.0211 (9)	0.0197 (8)	0.0207 (9)	−0.0022 (7)	0.0042 (7)	0.0041 (7)
C10	0.0165 (9)	0.0138 (7)	0.0233 (9)	0.0007 (6)	0.0002 (7)	−0.0008 (6)
C11	0.0275 (10)	0.0178 (8)	0.0164 (9)	−0.0017 (7)	0.0038 (7)	−0.0023 (7)
C12	0.0243 (9)	0.0164 (8)	0.0168 (9)	−0.0011 (7)	0.0051 (7)	0.0016 (6)

### Geometric parameters (Å, °)

**Table:**

Br1—C10	1.8944 (17)	C4—H4A	0.9900
S1—C1	1.6638 (18)	C4—H4B	0.9900
O1—C6	1.230 (2)	C5—H5A	0.9800
N1—C6	1.355 (2)	C5—H5B	0.9800
N1—C1	1.435 (2)	C5—H5C	0.9800
N1—H1	0.896 (5)	C6—C7	1.494 (2)
N2—C1	1.325 (2)	C7—C8	1.389 (2)
N2—C4	1.474 (2)	C7—C12	1.398 (2)
N2—C2	1.479 (2)	C8—C9	1.390 (2)
C2—C3	1.511 (3)	C8—H8A	0.9500
C2—H2A	0.9900	C9—C10	1.383 (3)
C2—H2B	0.9900	C9—H9A	0.9500
C3—H3A	0.9800	C10—C11	1.384 (2)
C3—H3B	0.9800	C11—C12	1.389 (2)
C3—H3C	0.9800	C11—H11A	0.9500
C4—C5	1.513 (3)	C12—H12A	0.9500
			
C6—N1—C1	120.52 (14)	C4—C5—H5A	109.5
C6—N1—H1	122.0 (15)	C4—C5—H5B	109.5
C1—N1—H1	115.4 (15)	H5A—C5—H5B	109.5
C1—N2—C4	120.85 (14)	C4—C5—H5C	109.5
C1—N2—C2	123.33 (14)	H5A—C5—H5C	109.5
C4—N2—C2	115.79 (15)	H5B—C5—H5C	109.5
N2—C1—N1	114.24 (15)	O1—C6—N1	121.04 (16)
N2—C1—S1	126.53 (13)	O1—C6—C7	121.79 (16)
N1—C1—S1	119.20 (13)	N1—C6—C7	117.11 (15)
N2—C2—C3	112.43 (15)	C8—C7—C12	119.34 (16)
N2—C2—H2A	109.1	C8—C7—C6	117.74 (15)
C3—C2—H2A	109.1	C12—C7—C6	122.84 (16)
N2—C2—H2B	109.1	C7—C8—C9	120.67 (16)
C3—C2—H2B	109.1	C7—C8—H8A	119.7
H2A—C2—H2B	107.8	C9—C8—H8A	119.7
C2—C3—H3A	109.5	C10—C9—C8	118.91 (16)
C2—C3—H3B	109.5	C10—C9—H9A	120.5
H3A—C3—H3B	109.5	C8—C9—H9A	120.5
C2—C3—H3C	109.5	C9—C10—C11	121.67 (16)
H3A—C3—H3C	109.5	C9—C10—Br1	119.22 (13)
H3B—C3—H3C	109.5	C11—C10—Br1	119.11 (13)
N2—C4—C5	111.95 (15)	C10—C11—C12	119.01 (16)
N2—C4—H4A	109.2	C10—C11—H11A	120.5
C5—C4—H4A	109.2	C12—C11—H11A	120.5
N2—C4—H4B	109.2	C11—C12—C7	120.39 (16)
C5—C4—H4B	109.2	C11—C12—H12A	119.8
H4A—C4—H4B	107.9	C7—C12—H12A	119.8
			
C4—N2—C1—N1	177.55 (14)	N1—C6—C7—C8	−173.35 (16)
C2—N2—C1—N1	−0.4 (2)	O1—C6—C7—C12	−167.57 (18)
C4—N2—C1—S1	−0.6 (3)	N1—C6—C7—C12	9.7 (2)
C2—N2—C1—S1	−178.51 (14)	C12—C7—C8—C9	0.3 (3)
C6—N1—C1—N2	87.2 (2)	C6—C7—C8—C9	−176.75 (16)
C6—N1—C1—S1	−94.57 (18)	C7—C8—C9—C10	0.4 (3)
C1—N2—C2—C3	82.9 (2)	C8—C9—C10—C11	−0.5 (3)
C4—N2—C2—C3	−95.12 (19)	C8—C9—C10—Br1	179.13 (13)
C1—N2—C4—C5	92.2 (2)	C9—C10—C11—C12	0.0 (3)
C2—N2—C4—C5	−89.7 (2)	Br1—C10—C11—C12	−179.64 (13)
C1—N1—C6—O1	−5.7 (3)	C10—C11—C12—C7	0.7 (3)
C1—N1—C6—C7	176.95 (15)	C8—C7—C12—C11	−0.8 (3)
O1—C6—C7—C8	9.3 (3)	C6—C7—C12—C11	176.06 (17)

### Hydrogen-bond geometry (Å, °)

**Table:**

*D*—H···*A*	*D*—H	H···*A*	*D*···*A*	*D*—H···*A*
N1—H1···O1^i^	0.90 (1)	2.02 (1)	2.882 (2)	162 (2)

Symmetry codes: (i) *x*, −*y*+3/2, *z*−1/2.

## References

[bb1] Allen, F. H., Kennard, O., Watson, D. G., Brammer, L., Orpen, A. G. & Taylor, R. (1987). *J. Chem. Soc. Perkin Trans. 2*, pp. S1–19.

[bb2] Arslan, H., Flörke, U. & Külcü, N. (2003). *Acta Cryst.* E**59**, o641–o642.

[bb3] Arslan, H., Flörke, U. & Külcü, N. (2004). *Turk. J. Chem.* **28**, 673–678.

[bb4] Arslan, H., Flörke, U. & Külcü, N. (2007). *Spectrochim. Acta A*, **67**, 936–943. 10.1016/j.saa.2006.09.011 17049302

[bb5] Arslan, H., Flörke, U., Külcü, N. & Binzet, G. (2007). *Spectrochim. Acta A*, **68**, 1347–1355. 10.1016/j.saa.2007.02.015 17418631

[bb6] Arslan, H., Külcü, N. & Flörke, U. (2003). *Transition Met. Chem.* **28**, 816–819.

[bb7] Arslan, H., Külcü, N. & Flörke, U. (2006). *Spectrochim. Acta A*, **64**, 1065–1071. 10.1016/j.saa.2005.09.016 16455292

[bb8] Bruker (2002). *SMART*, *SAINT *and *SADABS* Bruker AXS Inc., Madison, Wisconsin, USA.

[bb9] El Aamrani, F. Z., Kumar, A., Beyer, L., Cortina, J. L. & Sastre, A. M. (1998). *Solvent Extr. Ion Exch.* **16**, 1389–1406.

[bb10] El Aamrani, F. Z., Kumar, A., Cortina, J. L. & Sastre, A. M. (1999). *Anal. Chim. Acta*, **382**, 205–231.

[bb11] Khawar Rauf, M., Bolte, M. & Anwar, S. (2009). *Acta Cryst.* E**65**, o249. 10.1107/S1600536809000051 PMC2968195 21581865

[bb12] Khawar Rauf, M., Bolte, M. & Badshah, A. (2009*a*). *Acta Cryst.* E**65**, o143. 10.1107/S1600536808041251 PMC2968059 21581602

[bb13] Khawar Rauf, M., Bolte, M. & Badshah, A. (2009*b*). *Acta Cryst.* E**65**, o240. 10.1107/S1600536809000063 PMC2968148 21581857

[bb14] Khawar Rauf, M., Bolte, M. & Rauf, A. (2009). *Acta Cryst.* E**65**, o234. 10.1107/S1600536808043444 PMC2968411 21581851

[bb15] Koch, K. R. (2001). *Coord. Chem. Rev.* **216**, 473-488.

[bb16] Özer, C. K., Arslan, H., VanDerveer, D. & Binzet, G. (2009). *J. Coord. Chem.* **62**, 266–276.

[bb17] Sheldrick, G. M. (2008). *Acta Cryst.* A**64**, 112–122. 10.1107/S0108767307043930 18156677

[bb18] Weiqun, Z., Baolong, L., Liming, Z., Jiangang, D., Yong, Z., Lude, L. & Xujie, Y. (2004). *J. Mol. Struct.* **690**, 145–150.

[bb19] Yuan, Y. F., Wang, J. T., Gimeno, M. C., Laguna, A. & Jones, P. G. (2001). *Inorg. Chim. Acta*, **324**, 309–317.

[bb20] Zhang, Y. M., Wei, T. B., Xian, L. & Gao, L. M. (2004). *Phosphorus Sulfur Silicon Relat. Elem* **179**, 2007–2013.

